# Sepsis as CNS-governed metabolic triage

**DOI:** 10.3389/fimmu.2026.1842915

**Published:** 2026-08-20

**Authors:** Barış Yurtsever, Demet Demirkol

**Affiliations:** 1 Department of Pediatrics, Istanbul Faculty of Medicine, Istanbul University, Istanbul, Türkiye; 2 Division of Pediatric Intensive Care, Department of Pediatrics, Istanbul Faculty of Medicine, Istanbul University, Istanbul, Türkiye

**Keywords:** active inference, cortisol, free energy principle, HPA axis, insulin signaling, metabolic shutdown, organ dysfunction, sepsis

## Abstract

Sepsis remains the leading cause of death in intensive care, yet decades of trials targeting individual inflammatory mediators have failed to reduce mortality. This failure may reflect a fundamental misunderstanding: sepsis may not be a disease of immune dysregulation but a centrally governed metabolic program, an evolved conservation response functionally analogous to bacterial sporulation. We bring together three lines of work. Two are established: the Selfish Brain Theory of hierarchical energy allocation and the Metabolic Shutdown Hypothesis of adaptive organ hibernation. The third is developed here from a preprint by one of the authors: the proposal that cortisol is a candidate driver of the septic state rather than an anti-inflammatory brake, and that, as a consequence, the systemic immune response can be read as an active-inference process governed by the central nervous system (CNS). These describe one phenomenon from different vantage points. The convergence yields what we call a sporulation-like conservation program: we propose that, when the CNS detects an existential threat, it activates a coordinated program of peripheral shutdown, resource centralization, and innate-dominant immune reconfiguration, mediated primarily through the hypothalamic-pituitary-adrenal axis. This response is adaptive when self-limiting but becomes pathological when the organism cannot terminate the program, a state we recognize as sepsis — specifically, the high-cortisol, catabolic, insulin-resistant sepsis phenotype to which this framework is limited. Drawing on the Free Energy Principle as an interpretive lens, we describe this governance as a pathological attractor state: the sporulation-like policy generates its own confirmatory sensory input, creating an immunometabolic echo chamber that leaves the CNS no reason to revise its model. Breaking this lock-in requires a signal the model cannot predict: a genuine metabolic surprise. We propose that insulin can serve as this termination signal, acting not through glucose control but as a CNS-directed anabolic signal that conveys metabolic safety. This reframing reinterprets the existing insulin trial literature and yields testable predictions, which we set out in full. This framework would shift the therapeutic objective from suppressing inflammation to providing the metabolic evidence the CNS needs to terminate its own emergency program, from fighting the response to persuading the controller.

## Introduction

Sepsis remains one of the leading causes of death in intensive care units worldwide (1), yet its fundamental pathophysiology presents a paradox that existing frameworks have not fully resolved. Organs appear to ‘fail’: kidneys cease filtering, lungs lose gas-exchange capacity, the heart weakens. Yet postmortem studies consistently reveal well-preserved tissue architecture with surprisingly little cell death. The degree of cellular injury observed is disproportionately small relative to the severity of clinical and biochemical organ dysfunction (2).

This discrepancy becomes more striking when recovery is considered. Survivors of multi-organ dysfunction typically regain function within days to weeks, even in organs with limited regenerative capacity, a timeline that is difficult to reconcile with significant structural damage and subsequent repair. Furthermore, tissue oxygen levels in resuscitated sepsis patients are frequently normal or even elevated, suggesting that classical ischemia-hypoxia models may not fully account for the observed dysfunction (3, 4).

Over the past three decades, the prevailing conceptual model has framed sepsis primarily as an immunological disorder: an excessive systemic inflammatory response (SIRS) followed by a compensatory anti-inflammatory phase (CARS). This framework has generated important insights and correctly identified key phenomena: systemic inflammation, cytokine release, and immune paralysis are well-documented features of the septic response (5). However, the consistent failure of therapies directly targeting these pathways to improve mortality raises a fundamental question: not whether these phenomena occur, but where they sit in the causal hierarchy (6–8). If inflammation and immune suppression are consequences of a deeper process rather than its primary drivers, then targeting them directly would be expected to yield exactly the pattern of results observed: transient biomarker improvement without meaningful survival benefit.

In this paper, we propose that sepsis-induced organ dysfunction may be better understood by reframing immunological phenomena as downstream manifestations of a more fundamental program: an ancient, conserved metabolic triage response governed by the central nervous system (CNS). By this, we mean that the systemic configuration of immunity and metabolism is centrally selected via the hypothalamic–pituitary–adrenal (HPA) axis and sympathetic output, rather than arising autonomously in the periphery. We term this process a sporulation-like metabolic program: a coordinated strategy that prioritizes vital organ survival by downregulating peripheral organ functions, functionally analogous to bacterial endospore formation under existential threat. Within this framework, we hypothesize that the inflammatory cascade and subsequent immune suppression are not errors or overreactions but predictable outputs of a centrally orchestrated resource reallocation program.

This hypothesis integrates three converging lines of evidence that have largely developed in parallel. The Selfish Brain Theory, advanced by Peters and colleagues (9), demonstrates that the brain actively prioritizes its energy supply through HPA axis activation and sympathetic-mediated suppression of peripheral glucose uptake. The Metabolic Shutdown Hypothesis, proposed by Singer and colleagues (10), suggests that organ dysfunction may represent an adaptive, hibernation-like reduction in cellular energy expenditure rather than structural injury. The third line is developed here from a preprint previously posted by one of the authors (11): the proposal that cortisol is better understood not as a compensatory anti-inflammatory brake but as a candidate driver of the septic state, and that, as a consequence, the systemic immune response can be modeled as an active-inference process governed by the CNS. (We reconstruct that argument below from the primary literature so that it stands independently of the earlier work.) Each of these lines captures a facet of what may be a single, integrated physiological program, one whose pathological persistence, we suggest, manifests clinically as sepsis and multi-organ dysfunction.

In what follows, we develop the theoretical foundation and evolutionary logic of this framework, examine the conditions under which this program becomes pathological, and explore the role of insulin not as a glucose-lowering agent but as a potential termination signal: an afferent input that the threat model cannot explain in its own terms and that therefore forces the CNS to revise that model and exit the program. We call the content such a signal must carry a metabolic safety signal: evidence that peripheral resources are available and being used, so that the conditions that justified the conservation program no longer hold.

## Theoretical integration

A note on terminology is warranted before we develop the argument. The framework we propose is not intended to apply to the entire, heterogeneous sepsis umbrella but to a specific clinical phenotype: the subgroup characterized by elevated cortisol, persistent catabolism, insulin resistance, an innate-dominant immune response accompanied by lymphosuppression, and reversible organ dysfunction. Throughout this paper, when we speak of the “septic state” or the “sepsis program” in the mechanistic sense of a CNS-governed metabolic triage, we refer to this phenotype and to the phase of illness in which the program is pathologically sustained. When we discuss epidemiological data and the clinical trial literature, however, we continue to use “sepsis” in its established, broad sense, since most of the studies we cite were conducted in heterogeneous, all-comers populations. The model we advance is therefore best understood as applying not to that population as a whole but to the definable subgroup within it.

### Redefining cortisol: from immunological brake to metabolic policy executor

Modern immunology has characterized cortisol as an immunosuppressant, an anti-inflammatory brake (12, 13). The logical basis for this is cortisol’s rapid and highly effective action on adaptive immunity, particularly on lymphocytes: many conditions in which excessive immune activity becomes pathological respond dramatically to corticosteroids. This well-documented effect is not in dispute. What we question is the next step: extending this adaptive-arm action to the immune system as a whole, labeling cortisol an immunosuppressant, and labeling any high-cortisol disease state an immunosuppressed one. This extension, we argue, creates serious problems.

Cortisol’s immune actions in fact run in two directions. On the adaptive arm, they are suppressive: lymphopenia, T-cell anergy, and reduced antigen presentation (12). On the innate arm, they are activating: prolonged neutrophil lifespan, neutrophil demargination, and increased marrow release, together producing neutrophilia (14–16); M2-dominant monocyte polarization (17); and expansion of the myeloid-derived suppressor cell (MDSC) compartment (18, 19). We therefore read cortisol not as a general immunosuppressant but as an adaptive-immunity suppressant.

The most important consequence of this distinction is that it releases high-cortisol states from the immunosuppression label. Foremost among them is the CARS phase of sepsis. The name itself encodes the assumption: a compensatory anti-inflammatory response mounted against a hyperinflammatory state, read as immune suppression or dysregulation (20–22). On our reading, CARS is not an anti-inflammatory state at all but an innate-dominant inflammatory one; high cortisol does not equal immunosuppression. It follows that the therapeutic goal cannot be to correct an immunosuppression, because what is present is not a suppression but a reallocation of resources toward the innate arm.

A second consequence, and to our mind the more important, concerns the fallback position. Confronted with the association between high cortisol and poor outcomes (23, 24), the prevailing view holds that CARS is an anti-inflammatory state that is merely insufficient: cortisol is high, but relatively low, because inflammation is so intense that even high cortisol falls short. This is precisely the original definition of critical illness-related corticosteroid insufficiency (CIRCI), a relative insufficiency (25). Subsequent work, however, has shown that CIRCI is not a relative shortfall of cortisol against demand. During critical illness the carrier proteins that bind cortisol, corticosteroid-binding globulin and albumin, fall early and remain low, and the binding affinity of corticosteroid-binding globulin is further reduced at inflamed sites, so that the free, bioactive cortisol fraction rises even when total cortisol is only modestly elevated; at the same time, hepatic and renal breakdown of cortisol is suppressed, slowing clearance and sustaining the high free fraction (26–28). This sustained high free cortisol exerts negative feedback on the axis, suppressing ACTH, and the adrenal cortex, deprived of its trophic stimulus, becomes impaired over time, so that cortisol production eventually falls. CIRCI, on this account, is not a relative insufficiency present from the outset but an acquired adrenal insufficiency, a late complication of sepsis, the very opposite of a state in which cortisol was ever in short supply.

Treatment with cortisol in sepsis has been tried repeatedly as the other side of the same coin, yet over more than two decades of randomized trials, it has produced one of the most inconsistent literatures in critical care (**Table 1**). The two trials reporting a survival benefit (29, 30) shared a distinctive design: hydrocortisone combined with fludrocortisone in patients with established, vasopressor-dependent septic shock. A further point is worth noting about these two trials: in the first (29), the reduction in mortality was confined to the subgroup of ACTH non-responders who received hydrocortisone. That is, in the low-cortisol patients now recognized as having CIRCI, a complication of sepsis. Another two trials, which found no mortality benefit, tested hydrocortisone alone (31, 32). Two further trials (excluded from **Table 1** because their design does not permit a clean steroid-versus-placebo mortality comparison) point the same way: HYPRESS (33) gave hydrocortisone to patients with severe sepsis before shock was established and did not reduce progression to shock (21.2% vs 22.9%) or mortality; and in the factorial VANISH trial (34), where mortality was a secondary endpoint, a transcriptomic reanalysis (35) found that exogenous steroid was associated with higher mortality specifically in the SRS2 endotype. Across this body of work, no reproducible survival effect emerges. The one finding these trials consistently share is faster resolution of shock (CORTICUS, 3.3 vs 5.8 days; ADRENAL, 3 vs 4 days; APROCCHSS, more vasopressor-free days, 17 vs 15), the expected acute action of cortisol on the circulation (26, 36), an effect on the hemodynamic output of the state rather than on its course. Pooled analyses point the same way. Meta-analyses of low-dose corticosteroids in vasopressor-dependent septic shock report faster shock reversal, with at most a short-term mortality signal concentrated in the most vasopressor-dependent patients (37, 38). This is the same effect seen in the individual trials: cortisol speeds the resolution of shock — an effect on the patient’s hemodynamics, not on the course of the disease. When early survival does improve, it improves only in the sickest, most vasopressor-dependent patients, in whom faster hemodynamic recovery most directly helps them survive the acute phase.

**Table 1:** Corticosteroid trials in septic shock and severe sepsis.

Trial (year)	Population (n)	Regimen	Mortality endpoint	Steroid (%)	Control (%)	P
Annane (2002) (29)	Septic shock (299)	HC + fludrocortisone	28-d, ACTH non-responders (n=229)	53.0	63.0	0.02
CORTICUS (2008) (31)	Septic shock (499)	HC	28-d, all patients	34.3	31.5	0.51
APROCCHSS (2018) (30)	Septic shock (1241)	HC + fludrocortisone	90-d, all patients	43.0	49.1	0.03
ADRENAL (2018) (32)	Ventilated septic shock (3658)	HC infusion	90-d, all patients	27.9	28.8	0.50

A study of a different design, one that might be read as supporting the use of corticosteroids in sepsis-like states, also deserves mention here. As its name indicates, CAPE-COD (Community-Acquired Pneumonia: Evaluation of Corticosteroids) (39) shows, in a methodologically sound trial, that hydrocortisone reduces mortality in community-acquired pneumonia: death occurred in 6.2% of the hydrocortisone group versus 11.9% of the placebo group at day 28 (absolute difference, -5.6 percentage points; 95% CI, -9.6 to -1.7; P = 0.006), and in 9.3% versus 14.7% at day 90. This trial nonetheless does not provide an argument against our claims, for two reasons. First, septic shock patients were excluded, and whether the remaining patients met criteria for sepsis is unknown, since the trial was built around a diagnosis of pneumonia rather than sepsis; they may or may not have had sepsis. Second, and more interesting, the group in which mortality was reduced, the hydrocortisone group, received considerably more insulin than the placebo group: among patients given insulin, the median daily dose by day 7 was 35.5 versus 20.5 IU/day (P < 0.001), and insulin was administered to 231 patients versus 177. As noted in the Introduction, insulin is the treatment we propose for sepsis in this paper. Because insulin exposure here was not randomized, this can only be a hypothesis-generating observation; it leaves open the question of whether insulin contributed to the reduction in mortality. By its design the trial cannot separate the two, since insulin was given to control hydrocortisone-induced hyperglycemia rather than as a treatment in its own right. We return to this observation below, in connection with the distinction between glucose-targeted and non-glucose-targeted insulin protocols; on that distinction the higher and sustained insulin exposure of the hydrocortisone group is consistent with the account developed here.

We believe the inconsistency that runs through the cortisol trials has a real solution: to redefine cortisol not as a brake but as a candidate driver of sepsis, reversing the direction of the causal arrow. And we believe a strong body of literature supporting that candidacy is already at hand. Three independent lines converge. First, when corticosteroids are administered from outside, they reproduce the same cellular profile of neutrophilia, lymphopenia, falling monocyte HLA-DR, MDSC expansion (40–42), so the signature can be manufactured and is not bound to illness severity. Second, the withdrawal direction points the same way: as cortisol is lost in CIRCI while the illness (sepsis) itself runs on undiminished, the immune profile reverses, with lymphocytosis and eosinophilia (43–45) replacing the lymphopenia and eosinopenia of the high-cortisol state. Third, and most directly, the path is mechanistic rather than correlative: the fall in monocyte HLA-DR that marks sepsis tracks cortisol rather than catecholamines or interleukin-10, proceeds through transcriptional downregulation of the class II transactivator, is reproduced *in vitro* by glucocorticoids, and is abolished when the glucocorticoid receptor is blocked (46). Manufactured from outside, reversed on withdrawal, and traceable through a defined receptor: on this evidence, the phenotype is more consistent with an effect of cortisol than with a mere correlate of severity.

Taken together (**Table 2**), cortisol emerges as a strong candidate for a driver of the septic immune phenotype rather than a marker carried alongside it.

**Table 2:** Lines of evidence bearing on cortisol as a candidate driver of the septic immune phenotype.

Observation	Source
Exogenous glucocorticoids reproduce the components of the phenotype: neutrophilia, MDSC expansion, monocyte HLA-DR loss, and lymphoid suppression	(40–42)
The fall in monocyte HLA-DR that marks sepsis tracks the high cortisol of the state rather than the catecholamines or interleukin-10 that might otherwise have explained it, and it occurs through transcriptional downregulation of the class II transactivator; *in vitro*, glucocorticoids reproduce this loss, and it is abolished when the glucocorticoid receptor is blocked	(46)
In CIRCI, where the glucocorticoid signal falls while illness (sepsis) severity persists, the eosinopenia and lymphopenia of the high-cortisol state give way to eosinophilia and lymphocytosis, a reversal that follows the falling signal rather than the persistent severity	(43–45)
Higher endogenous cortisol is associated with worse, not better, outcomes, which a compensatory role would not predict	(23, 24)
Raising cortisol does not produce a consistent survival benefit; the effect that recurs is faster shock reversal, an action on the circulatory output rather than on survival	(29–35)
The compensatory label rests on extending cortisol’s lymphoid-suppressive action to immunity as a whole; on the innate arm, where cortisol’s effect is amplifying rather than suppressive, this extension is not supported	This paper

**Figure 1:**
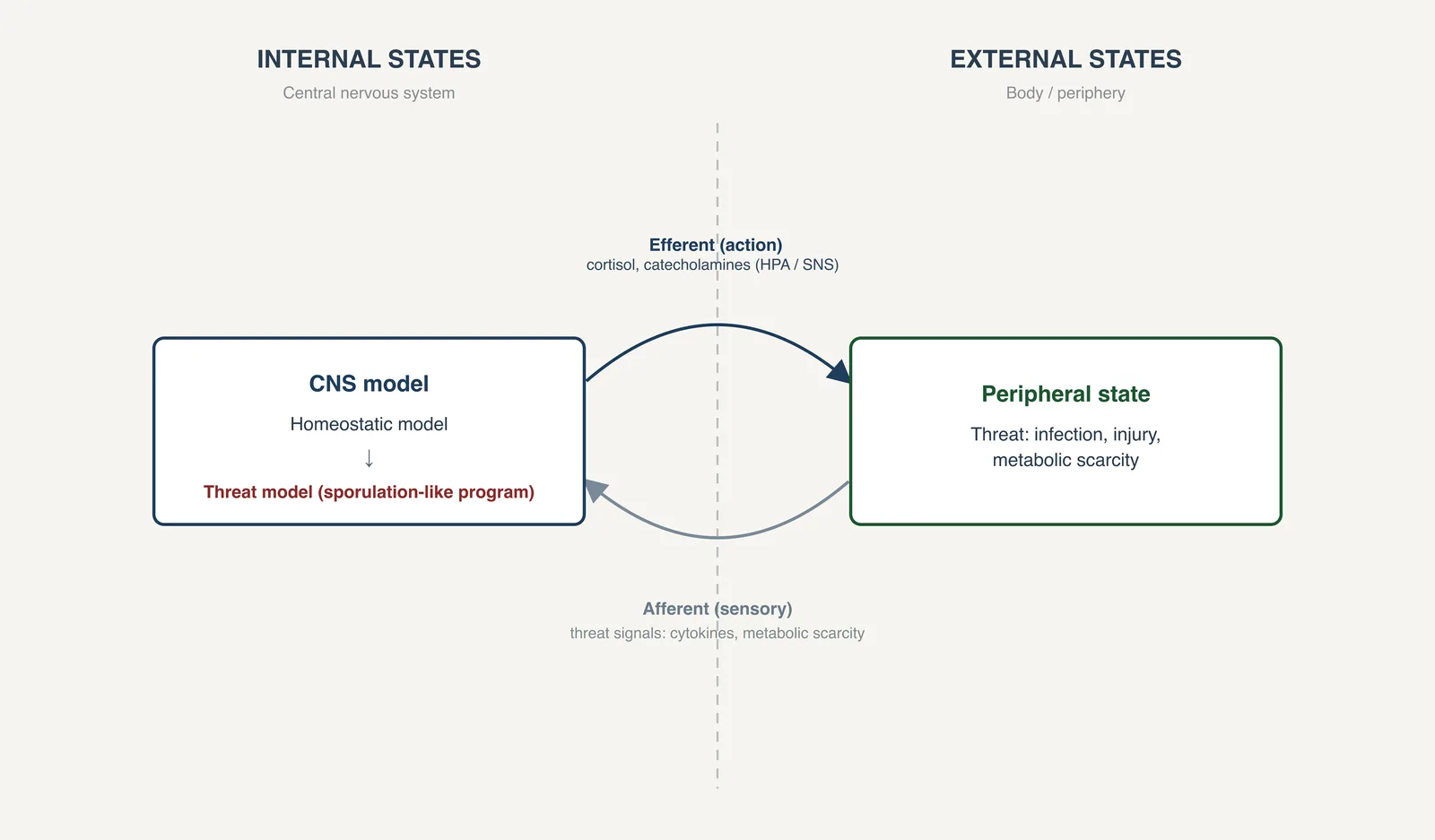
Sepsis as an active-inference loop governed by the CNS.

If we accept cortisol’s candidacy as a driver of sepsis, several logical inferences follow. Because cortisol output is governed by the HPA axis, a cortisol-driven immune phenotype is, by extension, a CNS-determined one: the immune configurations described above can be read as modes that the central nervous system selects rather than as autonomous immunological events. The two modes set out above separate cleanly. The high-cortisol state is innate-dominant, with the lymphoid arm suppressed; when CIRCI supervenes, and the cortisol signal is withdrawn while the illness persists, the system shifts into an adaptive-dominant mode marked by lymphocytosis. Further modes may well correspond to other conditions, but for the present argument we confine ourselves to this duality. To illustrate mode selection, we borrow the active inference framework from Friston (47) (**Figure 1**). The CNS (the internal states) cannot observe peripheral events directly; it infers them from a network of afferent signals arriving from the body (the external states). While those signals are consistent with the CNS’s current model, it keeps acting as before, the steady condition we call homeostasis. When signals of threat or scarcity fall outside the homeostatic model’s repertoire, the CNS engages a different model, one suited to threat, and issues a correspondingly different action. That action reshapes the periphery in the direction the organism intends, and so long as the returning afferent signals conform to what the threat model predicts, the model is not revised: the same action is reissued, and the threat model remains the CNS’s operative view of the world. In the language Levin (48) has applied to chronic disease, this self-sustaining configuration is an attractor state, or more simply a feedback loop. Once cortisol is positioned within it, this framing yields, in our view, a productive result.

If we posit a sepsis mode, that is, if the internal states constituting the CNS possess a dedicated mode for sepsis, then that mode must be defined by a specific set of afferent (input) and efferent (action) signals. The afferent signals have, in fact, been studied extensively and targeted therapeutically: most of the cytokines and related mediators implicated in sepsis have been manipulated in turn, yet no major benefit has emerged (8). One of the important efferent signals of sepsis, as this section has argued, is cortisol. Can we, then, alter the course of the disease by manipulating cortisol? Trials that have administered exogenous corticosteroids have failed to produce consistent results, while blocking cortisol is unlikely to fare any better, as the late septic complication CIRCI already shows us.

If neither afferent nor efferent signal manipulation alters the outcome, what then could the treatment of sepsis be? Active inference points to an answer. It describes a feedback loop in which a signal that the model cannot accommodate forces it to revise itself, and the revised model, in turn, generates its own actions. This line of reasoning suggests that the treatment of sepsis might take the form of a termination signal for the sepsis model, that is, an afferent signal the model cannot readily explain. A signal of this kind may be what is needed to prompt the model to revise itself. We turn to the question of what this signal might be in the sections that follow.

### The selfish brain: hierarchical energy allocation as the governing logic

We believe this framework offers one of the clearest answers to the question left open by the preceding section: if cortisol is not an immunosuppressant, then what is it?

The Selfish Brain Theory, developed by Peters and colleagues (9), fundamentally challenges the traditional glucocentric view of metabolism, the assumption that blood glucose is the primary regulated variable. Peters demonstrates that the brain occupies the apex of a metabolic hierarchy, actively controlling its own adenosine triphosphate (ATP) supply as the primary regulatory objective, with peripheral energy balance as a secondary objective.

The empirical foundation for this claim is striking: during caloric restriction and weight loss, the human brain mass is preferentially preserved while peripheral organs are catabolized to sustain it (49). This pattern indicates that the brain does not passively receive whatever glucose remains after peripheral consumption. Instead, it actively demands energy from the body through what Peters terms ‘brain-pull mechanisms’.

Two such mechanisms have been identified. Allocative brain-pull operates through the stress system: the brain activates the HPA axis and the sympathetic nervous system (SNS), causing cortisol and catecholamines to suppress peripheral insulin secretion and promote insulin resistance, thereby reducing glucose uptake by muscle and adipose tissue while simultaneously increasing hepatic glucose output (9, 50). The net effect is that glucose is redirected toward insulin-independent transport across the blood–brain barrier. Direct astrocytic brain-pull enhances glucose transport at the blood–brain barrier itself, ensuring preferential cerebral supply even under conditions of limited circulating glucose (9, 51).

The implications for critical illness are profound. Peters’ framework reinterprets insulin resistance not as a pathological process but as a mechanism, the brain’s tool for managing competition for peripheral glucose (50). From this perspective, the hyperglycemia and insulin resistance ubiquitous in sepsis are not errors to be corrected (52) but indicators of active CNS-driven resource reallocation.

We propose that sepsis presents us with precisely this configuration. When the brain receives a sensory pattern signaling an existential threat, it does not mount an isolated immune response; it mobilizes cortisol to execute a single, systemic program. The immunological arm of this program is the innate-dominant inflammatory response described above, not a failure of immune regulation but the deliberate reconfiguration of immune resources toward the innate compartment. As Peters and colleagues have shown, its metabolic arm operates as follows: the same cortisol signal that reshapes immunity also restricts peripheral glucose uptake and redirects energy toward the brain. Read this way, the immune and metabolic phenotypes of sepsis are not two coincident derangements but two outputs of one centrally issued instruction, with the same neuroendocrine signal (cortisol and sympathetic activation) serving as the common effector of both.

### The metabolic shutdown hypothesis: organ dysfunction as adaptive hibernation

If the CNS is actively reallocating energy to itself and reconfiguring immunity via cortisol, what happens to the peripheral organs that lose priority in this process? Singer and colleagues provide the answer (3, 10).

The Metabolic Shutdown Hypothesis proposes that sepsis-induced organ dysfunction is not caused by cellular destruction but instead represents a controlled, adaptive reduction in metabolic activity, a state analogous to hibernation or torpor observed across species under environmental stress (53). This hypothesis resolves several features of sepsis that are otherwise difficult to explain.

First, the histological paradox: despite severe clinical organ dysfunction, postmortem studies reveal surprisingly little cell death (2). Acute tubular necrosis, long assumed to underlie septic acute kidney injury, is in fact a misnomer: renal histology in sepsis exhibits minimal necrosis (54). Second, the oxygen paradox: tissue oxygen levels in septic organs are frequently normal or elevated (3), indicating that dysfunction cannot be attributed to inadequate oxygen delivery. Third, the recovery paradox: survivors of multi-organ dysfunction regain function within days to weeks, a timeline incompatible with the structural damage that requires repair and regeneration.

Carré and colleagues have demonstrated that recovery from sepsis-induced organ dysfunction correlates with mitochondrial biogenesis rather than cellular regeneration (55). This observation is consistent with a model in which cells downregulate their metabolic machinery (enter a hibernation-like state) and must rebuild it upon receiving signals that the threat has passed, rather than replacing destroyed tissue (10).

Within the framework we are constructing, metabolic shutdown is the peripheral consequence of the central decision. The CNS, via cortisol and sympathetic activation, redirects resources centrally and reconfigures immunity; the organs deprioritized in this process reduce their metabolic activity to survive on a diminished energy supply. This is not organ ‘failure’ in the traditional sense but organ ‘conservation’, a trade-off between function and survival at the cellular level, orchestrated from above (56).

### The cost of shutdown

This reading invites an immediate objection. If organs are conserved rather than destroyed, what accounts for the genuine injury and deaths that sepsis plainly causes? A shutdown of this scale cannot be without cost. Deprioritizing the periphery means that peripheral tissues stop doing the work they normally do, and tissue that stops working does not remain intact indefinitely: a proportion of cells will be injured or die, mitochondria are downregulated, some are damaged, endothelial function deteriorates, and the microcirculation is compromised by vasoconstriction and microthrombosis. Genuine structural injury, immune dysfunction, coagulation disturbance, and secondary pathogen-related damage are all part of the septic picture, and the framework does not dispute their presence. What it proposes is where they sit: not as the primary event but as the price of a centrally imposed withdrawal of function.

The decisive observation is that this price is disproportionately small relative to the clinical severity it accompanies. If the dysfunction of a failing organ reflected passive destruction, the histology should show destruction to match, yet it does not: cell death is limited, architecture is largely preserved, and survivors recover function on a timescale incompatible with structural repair (2, 54). A model of injury predicts damage proportional to dysfunction; a model of conservation predicts exactly this dissociation, in which profound functional shutdown coexists with modest structural loss. On this reading, the injury that does occur is the unavoidable cost of the shutdown rather than its cause. As the program persists, that cost accumulates, and an increasing fraction becomes irreversible, which is one sense in which a protective program, held too long, turns into the pathology we recognize as established multi-organ dysfunction.

The gut renders this logic concrete. The gastrointestinal tract does not operate in isolation; it functions in a symbiosis with its microbiota that depends on continuous work by the host epithelium, which maintains the mucosal barrier, secretes mucus and antimicrobial peptides, and supports the local immune interface (57). When the conservation program deprioritizes this tissue and the epithelium reduces its activity, one side of the symbiosis stops meeting its obligations, and the consequences are predictable: barrier integrity is lost, the microbial community is disturbed, and the boundary that normally separates the luminal flora from the host begins to fail (58). Here the cost of shutdown takes on a particular significance. The breakdown of the gut interface generates a fresh source of microbial and inflammatory signals, so that a measure taken to protect the organism becomes, through its own cost, a renewed threat reported back to the controller. We propose that this is one route by which the program sustains itself, supplying the CNS with continued evidence of danger and so resisting termination, a point we develop when we turn to why the program, once entrenched, fails to resolve. It also offers a reading, within this framework, of the long-standing observation that the gut behaves as a motor of multi-organ dysfunction in critical illness (59).

### Convergence: three perspectives on a single program

The three perspectives reviewed above have largely developed in parallel across different literatures. Yet they describe, from different vantage points, what may be a single integrated physiological program.

The first perspective reveals how cortisol affects the immune landscape: it shifts resources from adaptive to innate pathways, producing the clinical phenotype we argue is mislabeled as immunosuppression. Peters reveals why cortisol is deployed: it is the brain’s primary instrument for securing its energy supply when it is under threat. Singer reveals what happens to the periphery as a consequence: organs enter a hibernation-like state, trading function for cellular survival.

The program, viewed as a whole, operates as follows: when the CNS detects an existential threat via inflammatory and metabolic signals, it activates the HPA axis and the sympathetic nervous system, thereby releasing cortisol and catecholamines. These effectors simultaneously (a) redirect energy to the brain, (b) configure the immune system for a low-cost, innate-dominant posture, and (c) trigger metabolic downregulation in peripheral organs. The result is a coordinated survival response that preserves the organism’s core (the brain as the governing system, the adrenals as the effector apparatus) at the expense of peripheral function.

Seen this way, a third body of work falls into place. Once Peters’ metabolic model of SNS and cortisol action is also placed in the driving position for inflammation, the inflammatory reflex that Tracey and colleagues have mapped over more than two decades fits the picture like a missing piece. Tracey described a neural circuit through which the CNS senses and restrains inflammation: an afferent arc in which the vagus detects the molecular products of infection, and an efferent cholinergic arc in which vagal acetylcholine, acting through the α7 nicotinic receptor on macrophages, suppresses the release of TNF and other cytokines (60, 61). The claim, now well established, is that systemic inflammation is not autonomous but under reflexive central control, with its magnitude set centrally around a point the HPA axis can shift. This is precisely the governing architecture our reading of cortisol requires. Tracey also proposed that the vagus senses inflammation through the same kind of mechanism by which it senses metabolic variables: just as glomus cells signal the brain when oxygen falls, paraganglionic cells adjacent to the vagus appear to bind IL-1 and relay its presence centrally. Inflammatory and metabolic signals, on this account, are read through a common afferent architecture, exactly what our framework requires of a controller that does not perceive the threat directly but infers it from a pattern of signals.

The picture fits, with one exception: the cortisol arm. Tracey places cortisol on the same anti-inflammatory side as the reflex and grounds this in adrenalectomy: remove the adrenals, lose cortisol, and the inflammatory response is exaggerated. We read that observation differently, for the same reason CIRCI taught us to. The immune state produced by the loss of cortisol, whether through adrenalectomy or through CIRCI, is not the immune state of sepsis; they are not one and the same inflammation with the brake removed. What the cholinergic reflex inhibits is the innate arm, specifically macrophage cytokine release via the α7 receptor. What cortisol inhibits is the adaptive arm. These are suppressions of different things, and they cannot be grouped together as anti-inflammatory. Within the governing architecture Tracey established, which our hypothesis adopts, cortisol and the cholinergic reflex act on opposite arms of the immune system; only the reflex is anti-inflammatory in the sense Tracey intended.

This is not a new pathology but an ancient survival mechanism. In the sections that follow, we formalize this recognition as a hypothesis (the sporulation framework) and explore its evolutionary logic, mechanistic details, and the conditions under which it becomes pathological.

## The sporulation-like program

### The biological precedent: survival through strategic sacrifice

Across all domains of life, organisms confronting resource scarcity employ a remarkably consistent strategy: protect the essential core while sacrificing the periphery, and wait (62).

The bacterial endospore is perhaps the most striking example of this logic (63). When facing starvation or environmental extremes, Bacillus and Clostridium species do not attempt to maintain normal cellular functions as resources dwindle. Instead, they initiate a coordinated genetic program that packages essential material into a protected core while actively dismantling the vegetative cell (64). The resulting spore can persist for extended periods in a state of near-zero metabolism, reactivating only when environmental sensors detect that conditions have become favorable (65). Crucially, reactivation is not spontaneous; it requires specific external signals indicating that the threat has passed and resources are available.

This strategy, which we call ‘sporulation logic,’ embodies a fundamental survival principle: when available resources cannot sustain the entire system, survival depends on swift triage. The alternative (attempting to maintain all systems at reduced capacity) risks catastrophic failure of the entire organism, including the components essential for eventual recovery.

We suggest that mammalian physiology may maintain a functionally analogous program. The analogy is functional, not structural, and the difference defines it. Bacterial sporulation produces a discrete dormant body, a spore with near-zero metabolism that physically persists through the stressor; mammalian “sporulation,” as we use the term, produces no such structure. What carries over is the logic, not the form: a coordinated triage that protects a core by downregulating the periphery, without packaging that core into a dormant body. We use the term heuristically, for this shared logic of protected survival under scarcity, which is also why earlier work reached for the more cautious language of hibernation and dormancy. With that qualification in place: when the CNS detects signals indicating an existential threat under conditions of resource limitation, it appears to initiate a coordinated metabolic triage that reflects sporulation logic: consolidate resources into a minimal vital core, actively suppress peripheral organ functions, and sustain this state until appropriate signals indicate that the threat has passed and resources are sufficient for recovery. The theoretical integration presented above describes the mechanistic architecture through which this program may operate.

### The core-periphery architecture

If sepsis-induced organ dysfunction reflects a sporulation-like program, the system must distinguish between structures that are indispensable and those whose function can be temporarily sacrificed. We propose that the ‘core’ of the mammalian sporulation-like program must meet two criteria: its function cannot cease without causing immediate organismal death, and its ongoing operation is essential for eventual recovery.

Two structures clearly satisfy both criteria. The brain serves as the governing system—the entity that detects threats, selects responses, and manages the organism’s survival strategy. Critically, the brain also holds the termination switch: only the CNS can determine when the threat has passed and initiate recovery. Brain death is organismal death; there is no recovery without it (66). The adrenal glands serve as the effector apparatus. The adrenal cortex produces cortisol, which implements the metabolic triage policy, while the adrenal medulla releases catecholamines that execute the hemodynamic redistribution necessary to sustain core perfusion. Without continuous adrenal function, the survival program cannot be maintained (67).

The periphery, by contrast, exists on a continuum of deprioritization. Clinical and experimental observations suggest a rough hierarchy of sacrifice. Skeletal muscle is among the first to be catabolized, providing amino acid substrates for gluconeogenesis and acute-phase protein synthesis—a process that accelerates dramatically in sepsis (68). The splanchnic circulation undergoes selective vasoconstriction, redistributing perfusion centrally at the expense of the gut, a characteristic feature of the circulatory shock response (69). Renal function is downregulated, and septic acute kidney injury is increasingly understood as a functional rather than a structural phenomenon—tubular cells reduce metabolic activity rather than undergoing necrosis (54). Cardiac function is modulated by septic cardiomyopathy, which may represent a controlled reduction in myocardial energy expenditure rather than primary cardiac damage (70). Pulmonary function, while essential for gas exchange, is compromised as the innate immune program recruits activated neutrophils into the pulmonary vasculature, producing the clinical picture of acute respiratory distress syndrome (ARDS) (71).

This hierarchy is not rigid, and the precise ordering may vary depending on the nature and severity of the threat. What remains consistent, however, is the underlying logic: peripheral organ function is traded for cellular survival, with the degree of functional sacrifice roughly proportional to each organ’s dispensability during the acute crisis.

Adipose tissue provides a particularly visible illustration of this resource centralization. In chronic glucocorticoid excess, cortisol drives the accumulation of fat in central depots at the expense of the periphery, producing the characteristic centripetal adiposity of Cushing’s syndrome (72). This redistribution is cortisol-dependent and reversible: when hypercortisolism is corrected, central and truncal fat decrease while fat returns to the limbs, as Lönn and colleagues documented by imaging women with Cushing’s syndrome before and after curative treatment (73). That an equivalent centripetal redistribution is not observed in sepsis may simply reflect timescale: the hypercortisolemia of critical illness is unlikely to persist long enough to produce the changes that define Cushing’s syndrome.

### Evolutionary logic: species-level survival and individual cost

The sporulation-like program did not evolve to save every individual. Like any evolutionary strategy, it operates at the population level: if the response enabled six out of ten individuals to survive a crisis that would otherwise have killed all ten, it would be strongly selected for—despite being lethal to the remaining four. The thrombosis, vasoconstriction, metabolic shutdown, and immune reconfiguration that characterize the septic response are not design flaws; they are features of a program optimized for species persistence under ancestral conditions in which infections were self-limiting and medical intervention did not exist (62).

The patients who arrive in modern intensive care units are, in many cases, precisely those for whom this ancestral program is failing. Modern medicine can keep these patients alive through the acute crisis—antibiotics treat the infection, vasopressors maintain perfusion, and mechanical ventilation supports gas exchange. But keeping the patient alive is not the same as resolving the underlying program. We have altered the survival equation without addressing the mechanism producing the pathology (74).

### When the program becomes the pathology

The transition from adaptive response to pathological state occurs not because the program malfunctions, but because it fails to terminate. In the ancestral environment, termination was implicit: if the organism survived, the infection resolved, resources became available, and the metabolic signals reaching the CNS shifted from scarcity to sufficiency. Modern intensive care has disrupted this self-limiting cycle. We sustain patients through crises that would previously have been fatal, yet the CNS—still receiving signals consistent with ongoing threat—has no reason to exit the conservation program (75). The Persistent Inflammation, Immunosuppression, and Catabolism Syndrome (PICS) may represent precisely this state: patients who survive the acute insult but remain trapped in a sporulation-like program that runs indefinitely, neither recovering nor dying (76).

This analysis raises a question about current therapeutic practice. Standard sepsis management—vasopressors, exogenous catecholamines, and corticosteroids—may inadvertently replicate the very signals that sustain the program (77). Vasopressors mirror the hemodynamic redistribution the program executes through sympathetic activation; exogenous corticosteroids augment the cortisol-driven innate-dominant policy; permissive underfeeding reinforces scarcity signaling (78). In this sense, current supportive care may function as molecular mimicry of the sporulation-like program rather than as a termination signal.

A critical caveat is necessary here. This observation should not be taken as an argument against current supportive therapies. The sporulation-like program, even when pathological, has reorganized the entire organism around its execution. Abruptly withdrawing the effectors—removing vasopressors, withholding corticosteroids—would not terminate the program; it would collapse the hemodynamic and metabolic infrastructure on which the patient’s immediate survival depends. Furthermore, these same supportive therapies may prove essential as delivery infrastructure for any future termination strategy—for instance, maintaining adequate perfusion so that a termination signal can reach peripheral tissues.

**Figure 2:**
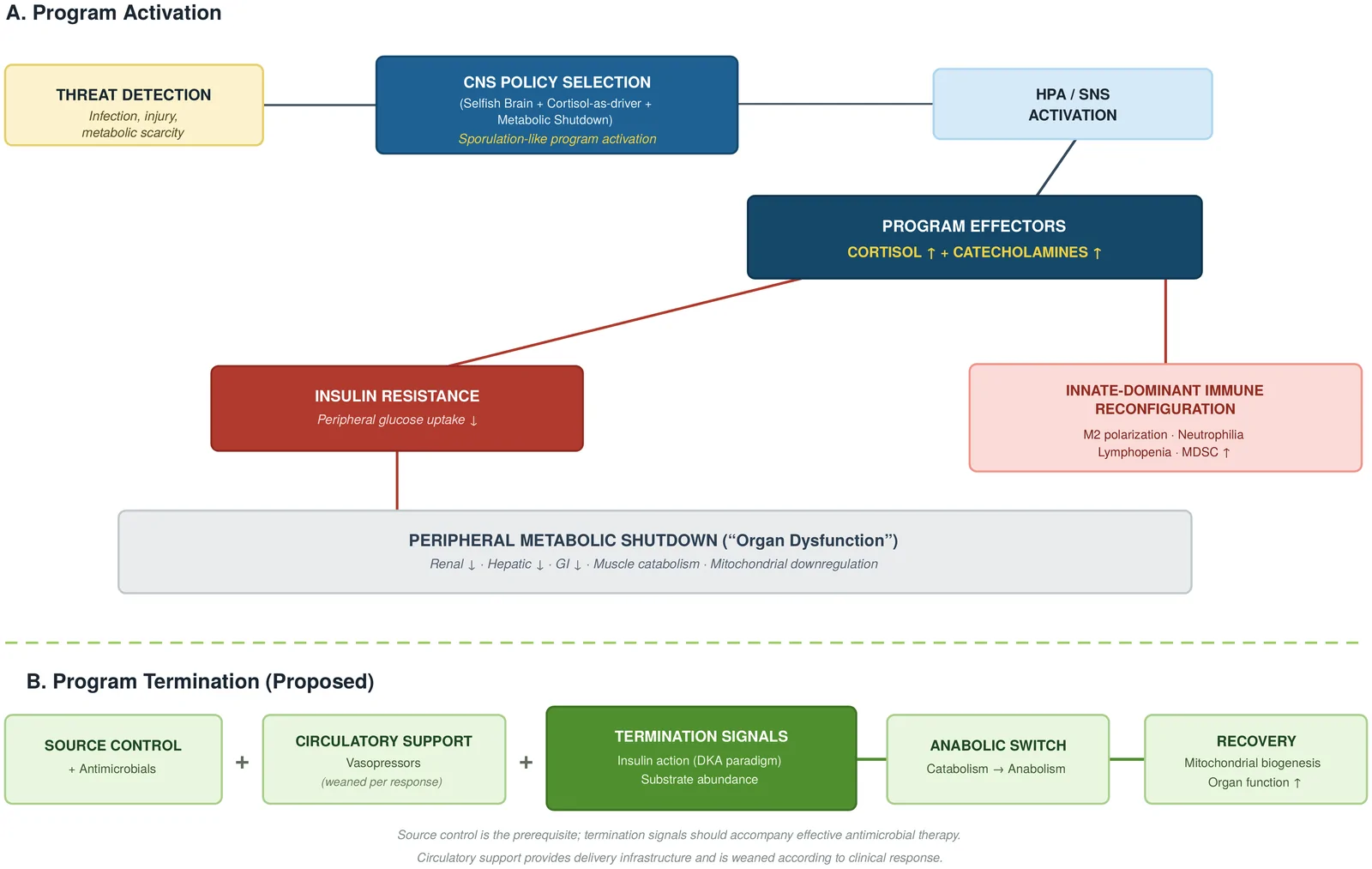
The sporulation-like program: CNS-governed metabolic triage in sepsis. **(A)** Program Activation. Following threat detection (infection, injury, or metabolic scarcity), the CNS initiates a coordinated conservation program. This program is executed via HPA/SNS activation, producing elevated cortisol and catecholamines as program effectors. These effectors drive two parallel outcomes: insulin resistance (restricting peripheral glucose uptake to centralize energy supply) and innate-dominant immune reconfiguration (M2 macrophage polarization, neutrophilia, lymphopenia, and MDSC expansion). The metabolic arm leads to peripheral metabolic shutdown—the clinical phenotype recognized as “organ dysfunction”—characterized by renal, hepatic, gastrointestinal, and muscular dysfunction, with mitochondrial downregulation. Critically, in this model, this shutdown is proposed to represent an active, CNS-directed sacrifice of peripheral function rather than passive organ damage. **(B)** Program Termination (Proposed, Hypothetical). Recovery requires three concurrent elements. Source control (antimicrobials and surgical intervention) is the prerequisite because the conservation program exists to protect the organism from a genuine threat that must be addressed first. Circulatory support (vasopressors) provides the hemodynamic infrastructure needed to deliver termination signals and is weaned based on clinical response. Termination signals—principally insulin delivered using the diabetic ketoacidosis (DKA) paradigm (continuous infusion with glucose co-management) alongside substrate abundance—communicate metabolic safety to the CNS, initiating an anabolic switch from catabolism to anabolism and ultimately enabling recovery through mitochondrial biogenesis and restoration of organ function. In this image, panel **(A)** integrates established endocrine and immune findings with their proposed interpretation as a single CNS-governed program, whereas panel **(B)**—which includes insulin as a termination signal—is hypothetical and has not been clinically validated.

What we propose, therefore, is not the withdrawal of current therapies but a reframing of the therapeutic objective. Rather than supporting only the body’s crisis response, the goal should shift to providing the CNS with a clear termination signal—one that communicates metabolic safety and resource availability. As the program begins to resolve in response to this signal, supportive therapies such as vasopressors may be gradually weaned based on clinical response. The question then becomes: what is this termination signal, and how might it be delivered? The complete model, including program activation and the proposed termination mechanism, is illustrated in **Figure 2**.

## Insulin as a termination signal

### Reframing insulin: from glucose-lowering agent to metabolic safety signal

Why insulin, of all possible interventions? The framework calls for a termination signal: an afferent input the CNS threat model cannot generate on its own, one that carries evidence of metabolic safety and thereby removes the justification for the conservation program. Insulin is the endogenous signal that carries precisely this content. It is the body’s principal fed-state anabolic signal, the physiological announcement that nutrients are abundant and that peripheral tissues can invest in growth and storage rather than conserve. That this candidate already exists in every intensive care unit, with well-characterized pharmacology and an established protocol for safe delivery, makes it not only the theoretically indicated signal but also a clinically testable one.

If correct, the sporulation framework demands a fundamental reconceptualization of insulin’s therapeutic role in critical illness. In the prevailing paradigm, insulin is administered to lower blood glucose—hyperglycemia is framed as the problem, and normoglycemia as the solution. This has led to ‘glycemic control’ protocols in which insulin is titrated to glucose targets, and the dose is reduced or stopped when glucose falls (79, 80).

We propose that this framing inverts the therapeutic logic. Within the sporulation framework, insulin resistance in sepsis is not a metabolic error to be corrected but a CNS-directed mechanism—the brain’s tool for restricting peripheral glucose uptake and centralizing energy supply (9). On this view, hyperglycemia is a consequence of this centralization rather than a primary pathology (52). Insulin’s therapeutic value, therefore, lies not in its glucose-lowering effect but in its capacity to override this centralization command.

This reconceptualization has a critical practical implication: the insulin dose needed to overcome pathological insulin resistance and deliver an adequate metabolic safety signal may far exceed the dose required to normalize blood glucose. If insulin is titrated to glucose targets, we confine ourselves to whatever dose achieves normoglycemia—which may be well below the dose required for effective signaling. On this view, insulin is the therapeutic agent, and glucose reduction is a side effect to be managed rather than the objective that governs dosing.

A clarification of terms is required at this point, since glucose-targeted is easily misread. Any insulin strategy must monitor and defend the glucose level, exactly as it must defend potassium and phosphate, and the strategy proposed here includes such limits and stopping rules; insulin therapy indifferent to glucose would be indefensible. What we mean is a protocol in which the dose itself is subordinated to a glucose target, so that the infusion is reduced or discontinued whenever glucose falls, making sustained signaling impossible by design. The claim we defend is therefore not that insulin delivered under glucose monitoring cannot affect outcome; it is that protocols which withdraw insulin in order to defend a glucose target have never tested sustained signaling. Read in this light, the CAPE-COD observation noted earlier is at least compatible with this account: hydrocortisone-induced hyperglycemia may have supplied a substrate margin permitting insulin to be infused with fewer interruptions, an unplanned analogue of the dextrose co-management we propose; what the trial itself reports is only that the hydrocortisone group received higher daily doses of insulin during the first week of treatment. The insulin protocol of that trial is not reported in sufficient detail to establish this, and the frequency of hypoglycemia and of infusion interruptions is unknown, which is why we advance the observation as hypothesis-generating rather than as evidence for the proposal.

### The DKA paradigm

This approach to insulin is not without precedent. Diabetic ketoacidosis (DKA) management embodies precisely the logic we propose (81). In DKA, the therapeutic goal is not glucose normalization but the reversal of a pathological metabolic state characterized by ketogenesis, lipolysis, and acidosis. The treatment paradigm reflects this: insulin is infused continuously at a fixed rate, and when blood glucose falls below a threshold, dextrose is co-administered to permit ongoing insulin delivery. The insulin does not stop; the glucose is managed to allow continued insulin action (82, 83).

The key principles of DKA management provide a conceptual template for the sporulation framework. First, insulin is the therapeutic agent, administered continuously regardless of glucose level. Second, glucose is managed separately—not as the target but as a safety parameter. Third, permissive hyperglycemia is accepted: glucose targets of 10–14 mmol/L are maintained while insulin continues its metabolic work. Fourth, aggressive electrolyte monitoring and replacement are mandatory, because high-dose insulin drives potassium and phosphate intracellularly (81). This infrastructure—continuous insulin infusion with glucose co-management—already exists in every intensive care unit and could serve as a theoretical starting point for designing sepsis-specific protocols in future mechanistic studies and trials, rather than a protocol to be transferred directly.

A clarification is in order. This protocol remains unproven and untested in septic patients and cannot be applied directly at the bedside. Moreover, we do not claim that the DKA protocol transfers to sepsis unchanged, nor that the two states share a pathophysiology. DKA (in its classic type 1 form) is an absolute insulin deficiency, corrected by replacing the missing hormone; sepsis is the opposite, an active, centrally driven resistance that insulin must overcome. We borrow DKA not as a pathophysiological equivalent but as the safest established framework for delivering high-dose insulin, with structured glucose co-management and electrolyte correction. Its glucose targets, and probably its electrolyte regimens, would need reformulation for sepsis.

### Reinterpreting the insulin trial literature

The history of insulin therapy in critical illness has been marked by apparent contradictions. Early enthusiasm following the landmark Leuven I trial gave way to caution after NICE-SUGAR and other studies showed harm from tight glycemic control (**Table 3**). The field has largely concluded that intensive insulin therapy is dangerous and that moderate glucose targets are preferable. We suggest that this conclusion, though understandable, may stem from a fundamental misinterpretation of what these trials actually tested.

**Table 3:** Major insulin therapy trials in critical illness and sepsis.

Trial	Year	Population	Intensive target	Conventional target	Key findings
Leuven I (79)	2001	Surgical ICU (n=1548)	4.4–6.1 mmol/L	10–11.1 mmol/L	43% reduction in ICU mortality (4.6% vs 8.0%); greatest benefit in prolonged ICU stays
Leuven II (84)	2006	Medical ICU (n=1200)	4.4–6.1 mmol/L	10–11.1 mmol/L	No overall mortality benefit; reduced morbidity; benefit in ICU stay ≥3 days
VISEP (85)	2008	Severe sepsis (n=537)	4.4–6.1 mmol/L	10–11.1 mmol/L	Stopped early; no mortality difference; severe hypoglycemia 17.0% vs 4.1%
Glucontrol (86)	2009	Mixed ICU (n=1101)	4.4–6.1 mmol/L	7.8–10 mmol/L	Stopped early; no mortality difference; 8.7% vs 2.7% hypoglycemia
NICE-SUGAR (80)	2009	Mixed ICU (n=6104)	4.5–6.0 mmol/L	≤10 mmol/L	INCREASED mortality in intensive group (27.5% vs 24.9%); 6.8% vs 0.5% severe hypoglycemia
COIITSS (87)	2010	Septic shock (n=509)	4.4–6.1 mmol/L	<8.3 mmol/L	No mortality benefit; more severe hypoglycemia in intensive group
CGAO-REA (88)	2014	Mixed ICU (n=2684)	4.4–6.1 mmol/L	<10 mmol/L	No significant difference; higher hypoglycemia in intensive group

The Leuven I trial (79) demonstrated a remarkable 43% reduction in ICU mortality with intensive insulin therapy in surgical ICU patients. This protocol is notable for two features that distinguish it from what followed: very intensive monitoring, and adequate nutritional support through parenteral nutrition (89), which together allowed insulin to be delivered continuously without provoking dangerous hypoglycemia. A secondary analysis of the cohort attributed the benefit to glycemic control rather than to insulin dose (90); the trial was not designed to separate insulin exposure from glucose reduction; there too, insulin was titrated to a glucose target, so insulin exposure was not an independent variable. We therefore do not read Leuven I as having tested insulin as a signal. We note only that, of all the trials in this literature, it came closest to the conditions under which such a signal could be delivered, and it is the one that showed benefit.

Subsequent trials did not reproduce this mortality reduction. Leuven II showed no overall survival benefit, and VISEP, Glucontrol, and NICE-SUGAR found no benefit, with NICE-SUGAR showing increased mortality, alongside consistently higher rates of severe hypoglycemia in the intensive arms (**Table 3**). On the conventional reading, this sequence settles the question: intensive insulin therapy is unsafe, and moderate glucose targets are preferable.

These trials differ along many dimensions at once: population (surgical, medical, mixed, or septic), nutritional support, insulin dose, glucose target, monitoring intensity, and the frequency of hypoglycemia. Given so many differences, no single factor can be isolated as the cause of their divergent results, and the literature’s inconsistency is unsurprising. Yet beneath this variation, the trials share one feature, and it is that feature our framework treats as decisive.

What all of these trials have in common is that none was designed to deliver insulin. Each was designed to lower glucose. Insulin was titrated to a glucose target and reduced or stopped when glucose fell, so insulin exposure was not an independent variable, but a quantity allowed to vary in the service of glycemic control. A trial designed to test insulin as a signal would look different. It would resemble the management of diabetic ketoacidosis, where insulin is the agent and is infused continuously, while glucose is co-managed with dextrose so that delivery need not be interrupted (81). No trial in this literature has that design. The existing evidence therefore speaks to whether tight glycemic control, achieved with insulin, improves outcome. It does not speak to whether sustained insulin signaling, delivered with adequate substrate, does. These are different questions, and only the first has been asked. The distinction is one of protocol architecture, not of monitoring: what disqualifies these trials as tests of insulin signaling is not that they measured glucose, which any safe protocol must do, but that they withdrew insulin whenever glucose fell.

## Discussion

Sepsis is a heterogeneous syndrome (91, 92), and the framework we propose corresponds not to the entire umbrella but to a specific phenotype within it. Under a single label, sepsis encompasses critical illness states that are pathophysiologically distinct; transcriptomic subtyping and the distinctions along the axes of innate activation and lymphoid suppression clearly illustrate this heterogeneity. This follows from the fact that sepsis is fundamentally defined as immune dysregulation in response to an infectious agent—a definition that is inherently broad.

The model defined here—sepsis as CNS-governed metabolic triage—targets a specific subgroup within this broad distribution, namely the “high-cortisol phenotype” we described earlier. This phenotype is characterized by elevated cortisol, persistent catabolism, insulin resistance, an innate-dominant immune response with lymphosuppression, and reversible organ dysfunction. We contend that states whose pathophysiology, and therefore treatment, differ this markedly should be distinguished from one another, and that our framework is most meaningful within this subgroup. Whether this phenotype might ultimately warrant consideration as a distinct clinical entity in its own right remains to be tested.

In delineating the boundaries of the model, we wish to emphasize that this definition is not rigid but rather a framework to be applied on a case-by-case basis. In the presence of additional clinical factors, one or more of the features listed above may appear to be absent; for example, in a neutropenic patient who has received bone marrow–toxic chemotherapy, the innate-dominant immune profile may not be fully observable. Such situations should be evaluated on an individual basis. Nevertheless, patients to whom the proposed model applies would be expected to exhibit the core features described above in nearly all cases.

### The persistence of a failing paradigm

The dominant conceptual framework in sepsis research holds that organ dysfunction results from an excessive or dysregulated inflammatory response—a ‘cytokine storm’—and that modulating this response should improve outcomes. This framework has spurred an extraordinary volume of research. Yet, as Shapiro and colleagues have documented, decades of laboratory study and more than 100 clinical trials designed to suppress inflammation have failed to reduce sepsis mortality (8). Anti-TNF-α, anti-IL-1, anti-interleukin-6 (IL-6), toll-like receptor 4 (TLR4) antagonists, and numerous other anti-inflammatory strategies have consistently failed to demonstrate benefit in adequately powered trials (7, 8, 93).

What is perhaps most notable is not the failure itself but the field’s response to it. Rather than questioning the underlying premise, the prevailing interpretation has been that the right target, timing, or patient subgroup has not yet been identified. Shapiro and colleagues argue that the concept of hyperinflammation has become, in Kuhn’s terminology, an unfalsifiable paradigm—one that generates its own explanations for failure and resists revision in the face of contradictory data.

We do not suggest that inflammation is irrelevant to sepsis pathophysiology. However, we propose that the persistent failure of anti-inflammatory strategies may point to a more fundamental issue: inflammation in sepsis may be a downstream consequence of a centrally coordinated program rather than the primary driver of organ dysfunction. If so, targeting individual cytokines would be analogous to treating fever rather than the infection—addressing a manifestation rather than the mechanism.

### Source control: the program exists for a reason

A critical caveat must accompany any discussion of program termination. The sporulation-like response, as we have described it, is not a malfunction. It is a survival program that evolved to protect the organism during existential threats. The body does not sacrifice peripheral organ function without reason; it does so because an invading pathogen or an overwhelming insult makes this sacrifice the optimal survival strategy under resource constraints.

This has a direct therapeutic implication: signaling metabolic safety to the CNS while the underlying threat persists could be counterproductive or dangerous. If the infection is not controlled, prematurely terminating the conservation program would restore peripheral metabolic investment in an environment where those resources may be consumed by the pathogen or where the organism cannot yet afford the metabolic cost of recovery.

Effective antimicrobial therapy and source control therefore remain the absolute prerequisites for any termination strategy (94). The sporulation framework does not diminish the importance of antibiotics and surgical source control—it elevates them. In this model, source control is not merely one component of the treatment bundle; it is the precondition that makes program termination safe (94, 95). Insulin, as a termination signal, should be conceptualized as an intervention that follows or accompanies effective source control, not as one that replaces it. This proposal remains hypothetical and requires validation in preclinical models before clinical translation.

### Septic encephalopathy: evidence against or evidence for CNS governance?

A potential objection to the CNS governance model is that the brain itself appears to be affected in sepsis, with clinical manifestations including sepsis-associated encephalopathy. However, this observation is entirely consistent with the framework when one recognizes that the brain applies the same hierarchical logic internally. Septic encephalopathy selectively impairs cortical functions—consciousness, cognition, attention—while the subcortical structures essential for program governance are comparatively less affected. HPA axis activation intensifies rather than diminishes. Although autonomic and brainstem regulation are perturbed in sepsis, the neuroendocrine output and respiratory drive that sustain the conservation program are broadly maintained until late (96). The clinical picture of delirium progressing to stupor represents the progressive sacrifice of metabolically expensive cortical activity, conserving energy for the subcortical structures that execute the conservation program. This is consistent with the well-established concept of sickness behavior as an adaptive, centrally mediated response, with septic encephalopathy representing its extreme manifestation (97).

Upon closer inspection, the sporulation hypothesis can be read as an extension of sickness behavior, carried further and pushed to its limit. Dantzer and colleagues established, over several decades, that an organism mounting an immune response centrally suppresses costly activities it can do without, withdrawing energy from foraging, social engagement, and exploration so it can be spent on defense; the metabolic cost of an activated immune compartment can be met only if the rest of the organism consumes less (97, 98). What we propose extends both the direction and the targets of that reallocation. Where sickness behavior withdraws energy from behavior, the program we describe withdraws it from the peripheral organs themselves; and where sickness behavior is a temporary dialing-down of activity, the sporulation-like program is a deeper triage that sacrifices peripheral function to preserve a vital core. The sporulation hypothesis is this same conservation logic carried inward, from the level of behavior to the level of organ and cellular metabolism.

### Does the body make mistakes?

There is a tradition we share that declines to read the body’s responses as mistakes. The prevailing habit is to treat a reaction the organism should not, by today’s lights, mount as evidence that the organism is in error. We think this is the wrong starting point. An organism that has been selected over a very long evolutionary history to survive under conditions quite unlike the present one deserves the assumption that even a response that shortens its life today was shaped toward some end. We use end here in the sense of teleonomy, the apparent purposiveness that selection builds into a system, not any conscious intention.

Medzhitov, Schneider, and Soares offered one of the most productive expressions of this view with the concept of disease tolerance: the capacity to limit the damage an infection causes without reducing the pathogen burden, distinct from resistance, the elimination of the pathogen itself (99). We find the distinction persuasive and accept tolerance as a genuine defensive strategy. When a tolerance mechanism succeeds, the resulting state is no longer well described as pathology; it shades toward an accommodation between host and insult, much as a chronically acid-exposed esophagus that remodels into an acid-resistant epithelium is, whatever its later risks, an adaptation that buys survival the unremodeled tissue might not have had. A protective adaptation of the past can, of course, become the liability of the present, which is the same tension our framework presses elsewhere.

Our reading departs from this on where sepsis falls. We do not read sepsis as a tolerance state. In Medzhitov’s terms, it is closer to resistance, because the sepsis we have defined is not the host bearing an insult where it stands but a withdrawal into a narrowed compartment from which the invader is fought. What characterizes the septic state is an active narrowing of the defended territory: the regional redistribution of circulation that preserves the core at the expense of the splanchnic and peripheral beds, the thrombotic closure of the periphery, and the cortisol-driven mobilization of stored substrate toward central use. These are not the marks of a host tolerating damage in place; they are the marks of a center that, sensing its resources threatened, draws them into a smaller compartment and mounts its defense from there. Tolerance is the state the organism can hold while it can still afford to bear the insult where it stands; the sporulation-like program is what it falls back to when it can no longer do so.

## Predictions

A hypothesis of this kind is useful only to the extent that it can be shown to be wrong. We therefore state the framework’s central commitments as prospectively testable predictions, each paired with the observation that would count against it. The earlier reinterpretation of past trials is *post hoc* and serves to generate these predictions; the framework’s empirical standing rests on the prospective tests below, not on that reinterpretation.

We group the predictions into two tiers. The first focuses on mechanisms the framework treats as load-bearing, so a clear negative result would directly challenge the model. The second concerns readouts that track program resolution; these are expected to move with recovery and can corroborate the framework, but because each has multiple physiological determinants, none is decisive by itself.

### Core predictions

Prediction 1. As the cortisol signal fails in CIRCI, the immune phenotype should shift toward adaptive dominance, including a recovery of monocyte HLA-DR. If cortisol drives the innate-dominant, HLA-DR-low phenotype of sepsis rather than marking its severity, then the late decline of cortisol in CIRCI should release that suppression: monocyte HLA-DR should rise, even as illness severity persists.

Refutation: if monocyte HLA-DR falls further, or fails to recover, as cortisol declines in well-characterized CIRCI with severity held constant.

Prediction 2. Insulin delivered as a sustained signal, rather than titrated to a glucose target, should improve outcome in septic shock. In a defined septic shock population, continuous insulin uncoupled from a glucose target, with dextrose co-administration to sustain delivery and prevent hypoglycemia, should reduce 28-day mortality relative to conventional glucose-targeted insulin.

A concrete design follows. The specific values in this section are illustrative assumptions offered to make the proposal testable, not a clinical recommendation; they would require formal dose-finding and safety studies before any use. The glucose target might be set at 10 to 11 mmol/L (180 to 200 mg/dL): this matches the conventional arm of standard care, making the comparison clean, and it preserves a sufficient safety margin against hypoglycemia. In Arm A (standard care), insulin is stopped once glucose reaches this target. In Arm B, insulin is not stopped at the target; instead, a dextrose infusion is added to maintain insulin delivery. Fixing the insulin rate in advance, for example between 0.05 and 0.1 U/kg/h, is difficult because the dose required to overcome pathological insulin resistance varies from patient to patient; titrating the rate to the individual patient’s insulin resistance, as judged by the glucose response to insulin, is the more reasonable approach. Continuous monitoring and replacement of potassium and phosphate are mandatory, as they are indispensable to continuous insulin therapy. The vulnerable point of the approach is hypoglycemia, which must be prevented without exception: when glucose cannot be held with dextrose, insulin is reduced and, if necessary, stopped.

We use 28-day mortality as the primary endpoint. Because a mortality endpoint requires a large sample and may be underpowered in a first trial, the markers of program resolution set out below (Predictions 4 to 6) serve as secondary endpoints: they respond earlier and would corroborate a mortality effect where it is present or reveal one not yet visible in mortality where it is not.

Refutation: if Arm B confers no survival benefit despite achieving sustained insulin exposure without hypoglycemia.

Prediction 3. The harm observed in earlier intensive-insulin trials should be attributed to hypoglycemia, not to insulin itself. In our reading, the danger in those trials lay in delivering insulin without substrate, causing glucose to fall and the conservation program to be reinforced. Sustained insulin given with adequate dextrose and strict prevention of hypoglycemia should therefore not reproduce that harm.

Refutation: if high insulin exposure increases mortality even when hypoglycemia is rigorously prevented, the claim that the hazard lies in substrate scarcity rather than in insulin is not supported.

### Markers of program resolution

Prediction 4. Markers of program resolution should move early and together. Where the program is actively terminated, the indices that track resolution—SOFA trajectory, time to vasopressor independence, lactate clearance, and anabolic and mitochondrial-biogenesis markers—should improve earlier than under standard care.

Prediction 5. Recovery of monocyte HLA-DR should accompany successful program termination. As the program resolves and the cortisol drive recedes, monocyte HLA-DR should recover faster where the program is actively terminated than under standard care. We frame this as a downstream readout of resolution rather than a direct action of insulin.

Prediction 6. Metabolic recovery signaling should increase as the program resolves. A shift in the cortisol-to-DHEA ratio toward DHEA may accompany termination, consistent with a transition from conservation to recovery. This is the least settled of the readouts: the ratio is not a validated monitoring tool, and its lack of change would point to a limitation of the biomarker rather than of the framework.

We summarize these predictions, their measurable readouts, and their refutation criteria in **Table 4**.

**Table 4:** Predictions of the sporulation framework, their measurable readouts, and the criteria that would refute them.

#	Prediction	Measurable readout	Refutation criterion
1	As cortisol falls in CIRCI, the immune phenotype shifts toward adaptive dominance	Monocyte HLA-DR; lymphocyte and eosinophil counts, with severity held constant	HLA-DR falls further or fails to recover as cortisol declines while severity is constant
2	Insulin as a sustained signal, not titrated to glucose, improves outcome in septic shock	28-day mortality, Arm A vs Arm B	No survival benefit in Arm B despite sustained insulin exposure without hypoglycemia
3	Harm in earlier intensive-insulin trials tracks hypoglycemia, not insulin	Mortality vs hypoglycemia incidence in the substrate-supported arm	High insulin exposure increases mortality even when hypoglycemia is rigorously prevented
4	Markers of program resolution move early and together	SOFA trajectory, time to vasopressor independence, lactate clearance, anabolic and mitochondrial-biogenesis indices	Supporting marker; multiple determinants, not refuting on its own
5	Monocyte HLA-DR recovery accompanies successful termination	Monocyte HLA-DR time course, between arms	Supporting marker
6	Metabolic recovery signaling rises as the program resolves	Cortisol-to-DHEA ratio	Supporting marker; failure to move indicates a biomarker limit

### Limitations and future directions

Several important limitations of this framework must be acknowledged. First, the specific neural circuits through which the CNS selects metabolic policies remain undefined. We have described what the CNS appears to decide but not the precise mechanisms by which it does so. Second, the sporulation hypothesis is supported by convergent indirect evidence from multiple established frameworks but lacks direct experimental validation. Its predictions are testable, but the tests have not yet been conducted. Third, the therapeutic implications rest on theoretical reasoning and analogy with DKA protocols rather than on clinical trial data in sepsis. Fourth, sepsis is a heterogeneous syndrome, and not all patients may be equally responsive to metabolic counter-signaling. Patient selection criteria and biomarker development will be essential for any future clinical application. Fifth, the optimal timing and sequencing of a termination strategy relative to antimicrobial therapy and source control require careful investigation.

A clinical safety statement is essential and is made here without qualification. The insulin strategy proposed in this paper is a hypothesis intended to motivate controlled investigation. It is not a treatment recommendation, and nothing in this manuscript should be used to justify high-dose or signal-targeted insulin in septic patients outside a prospective clinical trial. The proposal carries real and well-documented hazards. Because it deliberately uncouples insulin dose from a glucose target, it would raise insulin exposure above the level permitted by glucose-targeted protocols. The NICE-SUGAR trial linked intensive glucose control and severe hypoglycemia to increased mortality. The specific risks include hypoglycemia, hypokalemia, and hypophosphatemia driven by intracellular electrolyte shifts; fluid and dextrose burden from co-administered substrate; and increased glycemic variability, each of which is independently associated with harm in critically ill patients. These hazards are precisely why the strategy must first be evaluated in trials equipped with continuous glucose and electrolyte monitoring, predefined stopping rules, and rigorous hypoglycemia prevention, as set out in the falsifiable design proposed in the Predictions section. Until such trials establish both safety and benefit, this framework should inform research questions only, not bedside practice.

A further hazard concerns the substrate itself. In murine models, Wang et al. found that anorexia was protective and that nutritional supplementation was detrimental in bacterial sepsis, with glucose necessary and sufficient for this effect, whereas the same supplementation protected against mortality in influenza infection and viral sepsis, where blocking glucose utilization was lethal (100). Because the strategy proposed here delivers dextrose alongside insulin, this finding must be taken seriously: it raises the possibility that the substrate required to sustain insulin delivery is itself harmful in bacterial infection. Two considerations bound the inference. First, in those experiments glucose was administered without exogenous insulin, and the harm was attributed to glucose-driven suppression of the fasting-adapted stress response rather than to insulin signaling; isolated glucose loading is therefore not a test of the present proposal, in which glucose is not the intervention but the substrate cover that permits insulin to be infused without interruption. Second, the direction of the effect reversed with pathogen class within the same study, indicating that substrate provision is not uniformly harmful but context-dependent. Whether dextrose co-administration under continuous insulin infusion reproduces the mortality increase seen with glucose alone is an empirical question, and it should be answered in preclinical models before any clinical trial of this strategy proceeds: a design comparing insulin with dextrose against dextrose alone and against fasting in bacterial sepsis would both address this safety concern and provide a direct test of the framework, since on our account it is sustained insulin signaling, not substrate delivery, that carries the effect.

These limitations notwithstanding, we believe the framework yields a coherent and testable research agenda. Immediate priorities include mechanistic studies examining the relationship between insulin dose, rather than glucose level, and clinical outcomes, while controlling for substrate provision; prospective evaluation of metabolic biomarkers, such as the cortisol-to-DHEA ratio, for treatment monitoring; and metabolic phenotyping of sepsis patients to identify those most consistent with the conservation program profile. Longer-term investigations should include protocol development for insulin delivery using DKA-style glucose co-management in sepsis and outcome trials designed to test the termination signal hypothesis directly.

## Conclusion

Sepsis-induced organ dysfunction has resisted therapeutic intervention for decades, despite enormous investment in anti-inflammatory strategies. We suggest that this resistance may stem from a fundamental misunderstanding: the field has approached sepsis as a condition to suppress rather than as a program to terminate.

This paper brings together three converging lines of work. Two are established frameworks: the Selfish Brain Theory of hierarchical energy allocation and the Metabolic Shutdown Hypothesis of adaptive organ hibernation. The third, developed here from the primary literature, reframes cortisol not as an anti-inflammatory brake but as a candidate driver of the septic state, and interprets the systemic immune response as a CNS-directed policy rather than an autonomous immunological event. Read together, these perspectives describe a single physiological program from different vantage points. In this model, sepsis-associated organ dysfunction is interpreted not primarily as organ damage from an immune system in disarray, but, in substantial part, as the coordinated sacrifice of peripheral function by a CNS that has determined that resources must be centralized for core survival. This reading does not exclude genuine structural injury; we propose that an active, centrally governed conservation program accounts for a major component of the dysfunction that current injury-based models leave unexplained.

Cortisol occupies a central place in this account. On the evidence assembled here, it behaves less like a passive marker of severity and more like a candidate effector through which the program is executed: exogenous administration reproduces the sepsis immune phenotype, endogenous elevation is associated with worse rather than better outcomes, and signaling intensity in transcriptomic subtypes marks the worst prognoses. We present this as a strong and testable causal hypothesis rather than a settled conclusion.

The therapeutic implication follows directly. If organ dysfunction is driven largely by a centrally governed conservation program, recovery may require not the suppression of downstream mediators but the delivery of a termination signal that communicates metabolic safety to the CNS. We propose that insulin, reconceived as a signaling molecule rather than a glucose-lowering agent and delivered using infrastructure already established for DKA management, is a candidate for this signal. This proposal is a hypothesis for controlled testing, not a recommendation for practice. Effective antimicrobial therapy and source control remain absolute prerequisites, since the program exists to protect the organism against a real threat.

Whether this framework will yield clinical benefit remains to be determined by carefully designed trials. What the past three decades have made clear, however, is that repeatedly targeting individual inflammatory mediators while ignoring the metabolic governance system that may coordinate the septic response has not produced the breakthrough patients and clinicians need.

## Data Availability

The original contributions presented in the study are included in the article. Further inquiries can be directed to the corresponding author.
